# Extending Cellulose-Based Polymers Application in Additive Manufacturing Technology: A Review of Recent Approaches

**DOI:** 10.3390/polym12091876

**Published:** 2020-08-20

**Authors:** Denesh Mohan, Zee Khai Teong, Afifah Nabilah Bakir, Mohd Shaiful Sajab, Hatika Kaco

**Affiliations:** 1 Research Center for Sustainable Process Technology (CESPRO), Faculty of Engineering and Built Environment, Universiti Kebangsaan Malaysia, Bangi 43600, Selangor, Malaysia; denesh.mohan@gmail.com (D.M.); teongzeekhai@gmail.com (Z.K.T.); afifahnabilahbakir@gmail.com (A.N.B.); 2 Department of Chemical and Process Engineering, Faculty of Engineering and Built Environment, Universiti Kebangsaan Malaysia, Bangi 43600, Selangor, Malaysia; 3 Kolej GENIUS Insan, Universiti Sains Islam Malaysia, Bandar Baru Nilai, Nilai 71800, Negeri Sembilan, Malaysia; hatikakaco@usim.edu.my

**Keywords:** 3D printing, 4D printing, additive manufacturing, cellulose, biopolymer

## Abstract

The materials for additive manufacturing (AM) technology have grown substantially over the last few years to fulfill industrial needs. Despite that, the use of bio-based composites for improved mechanical properties and biodegradation is still not fully explored. This limits the universal expansion of AM-fabricated products due to the incompatibility of the products made from petroleum-derived resources. The development of naturally-derived polymers for AM materials is promising with the increasing number of studies in recent years owing to their biodegradation and biocompatibility. Cellulose is the most abundant biopolymer that possesses many favorable properties to be incorporated into AM materials, which have been continuously focused on in recent years. This critical review discusses the development of AM technologies and materials, cellulose-based polymers, cellulose-based three-dimensional (3D) printing filaments, liquid deposition modeling of cellulose, and four-dimensional (4D) printing of cellulose-based materials. Cellulose-based AM material applications and the limitations with future developments are also reviewed.

## 1. Introduction

The world population increases by 227,400 people a day, and this situation increases the burden on the earth as the world population is expected to reach 10.74 billion by 2100, which can be extremely detrimental from an environmental perspective [1]. Due to the increase in population, intense research has been done to cope with the manufacturing demands; consequently, unsustainable production from non-renewable resources resulted in significant global pollution and climate changes [2]. Efforts have been taken in material research to manufacture durable materials with a lower tendency to degrade. However, with the high utilization of materials, the end-of-life of the materials has to be managed to conserve the environment. Only 10% of plastics are recycled, 60% is dumped in landfills, and 30% are unaccounted for, which can be discarded in any part of the environment, thus resulting in environmental issues [3]. By 2050, the plastic industry may need 20% of the crude oil supply to accommodate plastic production if the trend remains unchanged [4]. Thus, the UK government came up with the aim of zero avoidable waste 2050 to have sustainable production and improve the environmental condition [5].

The decrease in fossil fuel resources and the increase in plastic consumption drive the search for alternative resources and technologies for more sustainable and environmentally friendly plastic production. Sustainable plastic materials should be produced from renewable resources without damaging the environment, easily recycled, and biodegradable under certain environmental conditions with low energy consumption [6]. Biodegradable plastics and composites manufactured from renewable resources such as biomass are considered as the future materials that could replace polymers that are currently produced from petrochemical feedstock [7].

Lignocellulosic biomass, which presents in the form of discarded industrial waste and agricultural residues, represents the most abundant and sustainable resource available [8]. The main constituents in the lignocellulosic biomass are cellulose, lignin, and hemicellulose and the compositions differ for different types of biomass [9,10]. Cellulose and hemicellulose comprise polysaccharide chains, whereas lignin consists of irregular three-dimensional and branched phenolic polymers, and the material is also amorphous in nature [11,12].

Cellulose is known to be the most abundantly available component of biomass that covers up to 50 wt. % of lignocellulosic biomass [13]. Many studies have been carried out on cellulose as it is one of the most sustainable and renewable materials with novel applications to improve certain products. Various types of cellulose can be used to synthesize nanocellulose from the cellulose, such as cellulose nanofibrils (CNF), cellulose nanocrystals (CNC), and bacterial nanocellulose (BNC). These nanocellulose products vary in terms of properties, morphology, and crystallinity, depending on the extraction method and biomass used [14,15]. The biocompatibility and biodegradability of nanocellulose drive researchers to study the use of nanocellulose in wastewater treatment, packaging, drug delivery, and biosensors [16]. The utilization of cellulose fiber as the feedstock for injection molding has now expanded to AM, which is among the coveted industries in the world [17]. 

AM is the process of manufacturing materials layer by layer to fabricate precise three-dimensional (3D) models using data from computer-aided design (CAD) software [18]. Currently, various AM technology are widely adopted in industrial and domestic applications due to ease of product fabrication. Most applications are limited to smaller scales that limit waste production and energy consumption without the usage of bigger machines [19]. AM has expanded to various industries, including metal, ceramic, and medical applications, and the current focus of this technique is bioprinting cardiovascular application, which involves 3D-printed heart valves [20,21,22,23]. Various materials to be used in AM have been developed, such as polymers, metals, and composites; however, most of these materials are harmful to humans and the environment due to the release of volatile organic compounds [23,24,25,26].

Therefore, many naturally derived polymers are used in current studies for the preparation of scaffolds by 3D printing due to the large potential in biomedical applications, particularly the replacement and regeneration of cells, tissues, or organs [27,28,29]. Various studies have been conducted in this field using different formulations of collagens, alginates, and chitosan, as these materials are well-known natural sources of polymers [30,31,32]. AM of cellulose-based materials is a promising option due to the renewable source and low cost of extraction with lower environmental degradation.

In this manuscript, cellulose-based three-dimensional (3D) printing and four-dimensional (4D) printing materials are reviewed. Then, 3D printing technologies and developed materials are discussed. Next, mechanical and physical characterization of AM-fabricated parts are reviewed, in addition to the improvement done to meet the industrial application needs. Cellulose as a biopolymer product and its incorporation in 3D printing filaments are studied. Liquid printing of cellulose and the rheological changes done to ease the extrusion process and maintain the shape fidelity of the constructed part are discussed, with the emphasis given on the research conducted in the last five years. The contribution of cellulose-based 3D printing materials in biomedical engineering, electronic engineering, textiles, food, and food packaging industries are presented. 4D printing technology and the smart materials developed in recent times are discussed briefly. Cellulose-based smart materials and cellulose-based 4D printing materials are reviewed. Finally, the limitations and future trends of cellulose-based AM materials are also provided.

## 2. Polymer-Based Additive Manufacturing

### 2.1. Types of Additive Manufacturing Technologies

AM technologies are the essential part of the whole 3D printing, bridging the 3D models, materials, and final applications based on the products needed by the industry. Originally, 3D printers were used to produce one or two fast prototype models to help developers fix faults and change the product as a fast prototyping solution. Different technologies have been developed by varying the technique of printing product on the build platform and the materials used for printing. 

**Figure 1:**
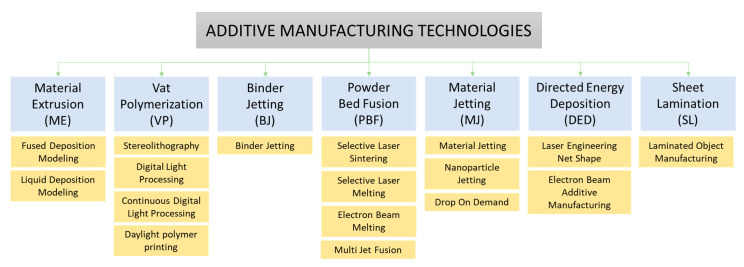
Additive manufacturing technologies category adapted from [33,34].

By referring to ASTM Standard F2792, the American Society for Testing and Materials (ASTM) has documented 3D printing technologies into seven categories, namely material extrusion, powder bed fusion, vat photopolymerization, direct energy deposition, binder jetting, material jetting, and sheet lamination, as shown in Figure 1 [33,34]. AM technologies of polymer-based materials are reviewed in Table 1.

### 2.2. Biodegradable Polymers for Additive Manufacturing

Polymer shows a major contribution in AM, whereby parts produced from the polymer are recorded with 51% contribution, 29% metal and polymer, and 19.8% is metal product [42,43]. Among the many available AM techniques producing 3D-printed polymers, FDM is mainly used for fabrication. FDM 3D print polymer has become an affordable technology. The cost of a commercial 3D printer in the market and the filament polymer is inexpensive. The two most common material in FDM are ABS and PLA, but these materials have their advantages and drawbacks that need to be solved. PLA printing is known for its green material, biodegradability, low warping, and good surface print; however, the method produces products with lower mechanical properties and low heat resistance, which affect their application in industries. Meanwhile, ABS products have better mechanical properties and thermal properties, but an unpleasant odor is produced during printing, which affects the health of the user and the environment [44].

**Figure 2:**
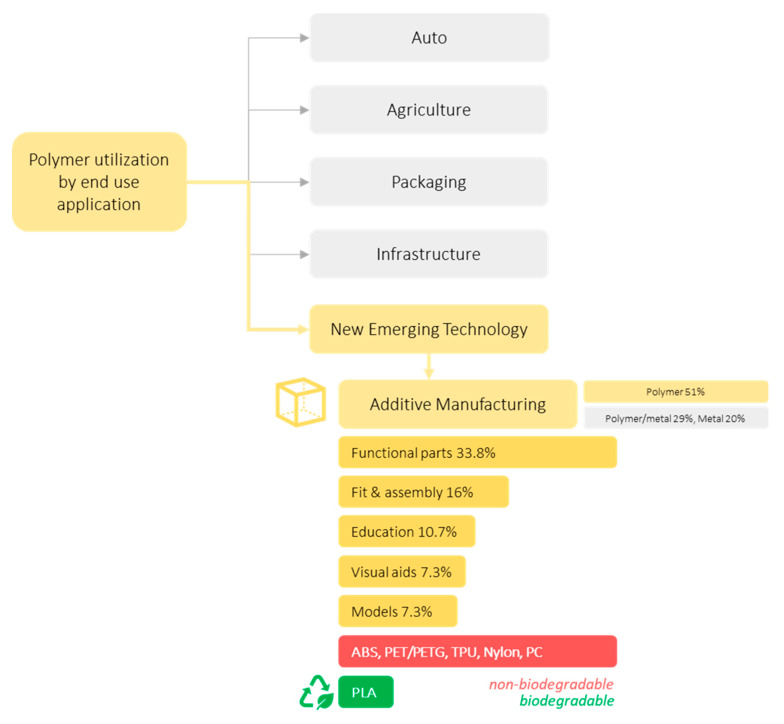
A new threat of plastic waste generation from additive manufacturing.

Due to the feasibility of this technology, it has been used as part of educational kits, prototypes, visual aids, and presentation models (see Figure 2). However, due to the lack of technical skills and quality of the 3D printer, the end-user tends to produce massive waste from supportive material, failed products, and broken plastic parts. The potential of AM as exploited technology to build the future is undeniable. However, this emerging technology has started making a new pathway of waste generation.

Over the last, the production of plastics has increased tremendously due to various types of applications mainly focusing on packaging, thus generating uncontrollable waste that degrades the environment. Most of the plastics used commercially are made from petroleum resources, one of the non-renewable sources of energy. Most of the used plastics are not managed well: most are dumped as waste, with only a small amount being recycled. For instance, about 1.5–4% or 5–13 million tonnes of plastic are dumped in the ocean every year [45]. The utilization of waste by the conversion to biopolymers is an effective approach of conserving the environment, and the practice is being focused on immensely in recent years. Biopolymers are defined as the naturally-derived polymers from living organisms or chemically synthesized from renewable matter, which can be used to replace conventional plastics [46]. According to ASTM D883, biodegradable plastic is defined as plastic degraded through degradation by naturally occurring organisms, such as bacteria, fungi, and algae [47].

Polymer biodegradation is a complicated process where many biological, chemical, and mechanical processes are involved in converting polymers to smaller molecular chains and then further converted to carbon dioxide and water [48]. Biodegradation occurs through three essential processes: biodeterioration, biofragmentation, and assimilation. Biodeterioration is the modification of physical and chemical properties polymer owing to the growth of microorganisms throughout the polymer surfaces. Biofragmentation is the breaking down of polymers into simpler structures, such as oligomers and monomers by microorganisms present. The final step is assimilation, where microorganisms are fed with necessary living sources from the breaking down of polymers, followed by the conversion of the carbon in the plastic to carbon dioxide, water, and biomass. Among polymer biodegradation factors are the chemical structure, the length of the polymer chain, the complexity of the polymeric formula, and the crystallinity of the polymer [49].

To introduce a biodegradable-based polymer in the 3D printing industry, modifications need to be intensively done so that polymers will be usable in various AM techniques. Many industries have been working on utilizing 3D-printed polymers to enhance the product properties by the addition of composites to have the desired properties. Thus, 3D printing of natural and synthetic polymers with composites has progressed immensely in recent years, together with the discovery of new materials. The challenges in 3D printing include the aspects from controlling the flowability to produce good extrusion, the mechanical properties, to the adhesion between layers in order to have a strong printed part. Despite these challenges, the complexity of the product and freeform fabrication are the driving factors to produce a 3D-printed product with less waste. Hence, since PLA in one of the biodegradable synthetic polymers, much attention is given to the study of the incorporation of different types of fillers (see Table 2) to improve the mechanical properties and the biodegradability of the commercially available polymers.

However, continuous study must be done to incorporate fillers at a higher percentage with the improved porosity and shrinkage for the developed materials to meet the required standards. The focus will be on the adaptation of 3D printing for industrial applications with the development of new 3D printing biodegradable-based polymer composites. Subsequently, the limited option of biodegradable filament in 3D printing can be catered by formulating biodegradable-based polymers as a new filament in the market. On the other hand, natural polymers such as chitosan, soy protein, and starch are also being studied intensively for biomedically applications owing to the good compatibility towards living cells for cell generations [50,51].

## 3. Cellulose-Based Polymers in 3D Printing Technology

**Figure 3:**
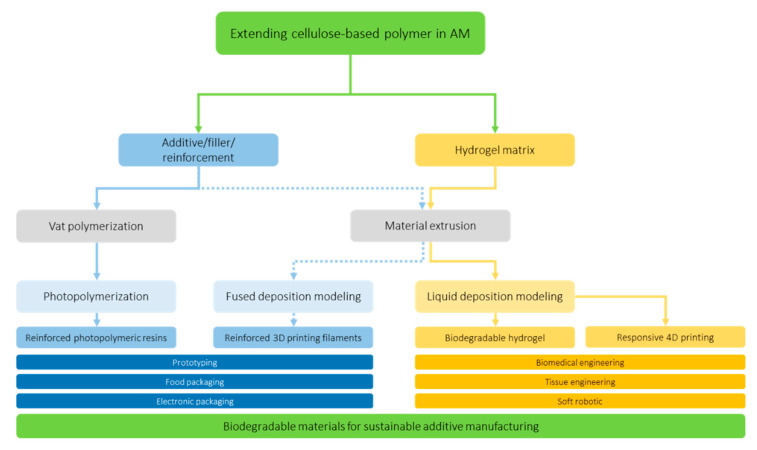
Possible pathway of extending cellulose-based polymer as green and biodegradable materials for sustainable additive manufacturing.

The main principles of both green chemistry and green engineering is focusing on the prevention of new generation waste. The prevention can be started in this new emerging technology by introducing sustainable and biodegradable materials in 3D printing applications. As early as possible, the polymer-based material used in the AM should pursue cradle to cradle design. On top of mechanical properties, the capability of cellulose-based polymer as bio-filler and hydrogel matrix will be a key to developing sustainable additive manufacturing (see Figure 3).

### 3.1. Cellulosic Biopolymer

Modification and reinforcement of cellulose into biopolymers are an ever-increasing trend in recent times owing to its biodegradability and low cost of extraction. Cellulose, which is a type of natural biopolymer, is the most abundant component present at the highest percentage in lignocellulosic biomass. Cellulose is a linear polysaccharide that consists of β-1,4-glycosidic bond connecting two β-d-glucopyranose with a degree of polymerization from hundreds to thousands [11]. Hydrogen bonds formed through the high number of hydroxyl groups and Van der Waals interaction are present on glucose rings, thus resulting in the crystallization of few cellulose chains into fibrils and structural regions of crystalline and amorphous regions, which give high mechanical properties and biocompatibility. Three hydroxyl groups present in one cellulose monomeric unit contribute to cellulose properties, such as surface hydrophilicity, biodegradability, and chirality [62]. Modifications can be done on cellulose due to the presence of hydroxyl groups to obtain cellulose derivatives through a number of reactions, such as esterification, etherification, and carboxymethylation [63].

Polymer composites reinforced with microcrystalline cellulose (MCC) have excellent properties, such as high tensile strength, low density, and different morphologies, but suffer from the issues of surface compatibility, moisture absorption, and poor wettability. MCC is a type of cellulose obtained from partial depolymerization of alpha-cellulose with acids, such as hydrochloric acid and sulfuric acid [64,65]. The amorphous phase of cellulose will be hydrolyzed, thus producing more crystalline and shorter fragments. The hydrolysis process parameters, including temperature, time, acid concentration, and fiber-to-acid ratio determine the mechanical properties of MCC. The reinforcement of MCC obtained from acid hydrolysis of wheat straw in the PLA matrix showed enhanced mechanical properties with an increment of tensile modulus and tensile strength by 27% and 8.4%, respectively [66]. Furthermore, the incorporation of MCC to concrete was studied owing to the high mechanical strength of MCC. The addition of MCC at 1 wt. % of cement in the concrete improved the buildability, as well as increased the compressive and flexural properties by 18.6% and 12.5%, respectively [67]. By looking at the trend of biocomposites, extensive research is being carried out using MCC to develop products for pharmaceutical, food, cosmetic, cement, and packaging material industries [68].

Carboxymethyl cellulose (CMC) is a type of cellulose derived with carboxymethyl groups (-CH_2_COOH) bonded to the hydroxyl groups of the cellulose monomer and often substituted with sodium to form sodium carboxymethyl cellulose. CMC is synthesized through the reaction with aqueous sodium hydroxide (NaOH) solution, followed by subsequent alkalization, carboxymethylation, and neutralization. The alkalization of CMC utilizes NaOH, carboxymethylation uses sodium chloroacetic acid at 60 °C, and neutralization uses ethanol at 96% concentration [69]. The increase of oxidized CMC percentage by four times in the CMC-CNF-xylan biocomposite managed to increase tensile strength by two times, and also improved tensile modulus [70]. CMC is commonly used in the pharmaceutical industry as a binder because CMC has the ability to form tough tablets, utilized for drug delivery and tissue engineering, and used in paper and textile industries [71,72].

Three types of nanocellulose (i.e., CNC, CNF, and bacterial cellulose (BC)) can be derived from cellulose through various methods, such as acid hydrolysis, high-speed homogenization, and bacterial process. Nanocellulose is being explored extensively owing to its high mechanical properties, low density, reinforcing properties, and biodegradability. CNC is derived from the isolation of crystalline regions present in cellulose with a combination of chemical and mechanical treatments. CNC can be viewed as stiff rod-like particles under the microscope; hence, it is known as cellulose whiskers or microcrystallites. Acid hydrolysis is a widely utilized method for the isolation of CNC from cellulose, and the highest yield of CNC depends on the optimum acid concentration of 56–60 wt. % and temperature of 60–70 °C, where high concentration and temperature will lead to the dissolution of cellulose [73]. Various methods have been considered in the isolation of CNC as a substitute to the acid hydrolysis method due to the difficult treatment of acid waste [74]. CNC possesses tremendously good properties such as high tensile strength, low density, optical transparency, and high surface area, which are suitable for many applications, such as biosensors, drug delivery, and water absorption [75]. The addition of 2 wt. % CNC in urea-formaldehyde adhesive for fabrication of medium-density fiberboard reduced formaldehyde emission by 17% and enhanced the mechanical properties of the fiberboard [76]. 

CNF is another type of nanocellulose isolated from cellulose through mechanical treatment. CNF can be produced using high-speed homogenization, producing web-like nanofibrils consisting of both crystalline and amorphous regions [77,78]. The high shearing force yields CNF having shear-thinning behavior with a high aspect ratio [79,80]. The electrospinning of CNF, PLA, and polybutylene succinate (PBS) can generate a composite with cell attachment and proliferation process that could be suitable for tissue regeneration applications with better mechanical properties and biodegradability due to the reinforcement by CNF [81]. The unique properties of CNF have been employed throughout various applications, such as food, drug delivery, electronic, tissue engineering, and cosmetic applications.

BC is a biodegradable nanocellulose synthesized through the microbial activity on cellulose. Oxidative fermentation of bacteria such as Gluconacetobacter, Sarcina, and Agrobacterium in non-synthetic or synthetic media will form cellulose fibrils with network-like structures that possess high surface area and mechanical properties [82]. Different bacteria can be used with the manipulation of culture conditions to synthesize BC in different shapes, such as gels and granules [83]. Surface grafting through the acetylation of BC to nullify the hydrophilicity of BC was able to disperse BC homogeneously in the PLA matrix and improve the stiffness of the nanocomposite while maintaining the tensile strength compared to the neat PLA [84]. Surface grafting through the acetylation of BC to nullify the hydrophilicity of BC was able to disperse BC homogeneously in the PLA matrix and improve the stiffness of the nanocomposite while maintaining the tensile strength compared to the neat PLA [83].

Cellulose acetate (CA) is one of the most valuable cellulose derivatives that is biodegradable and non-toxic. CA can be synthesized through the acetylation process by the reaction of cellulose with acetic acid and acetic anhydride using sulfuric acid as the catalyst [85]. The synthesized CA has excellent electrical conductivity, chemical, and thermal resistant properties that can be obtained with low cost, which encourages the use of CA in many applications, especially in the biomedical industry. The addition of 20 wt. % CA in the PVA matrix improved the porosity of the films, and the antibacterial activity of the composite was observed with the incorporation of vancomycin, which can be applied in wound healing applications [86].

### 3.2. Fused Deposition Modeling Filament

Cellulose particles in micro/nano size can be incorporated in 3D printing filaments to increase the mechanical properties of the printed products. This is due to the properties of cellulose, especially nanocellulose that possesses high surface area, high mechanical properties, and shear-thinning properties, thus making cellulose suitable for many applications. The 3D printing of cellulose has been mostly done using two approaches: filament extrusion and liquid deposition modeling. 

However, major issues arise in the compatibility of cellulose and polymer matrix due to the hydrophilicity of cellulose and hydrophobicity of the polymer matrix, in which cellulose requires surface modification to improve the compatibility and dispersion of cellulose in the polymer matrix. One of the earliest surface grafting methods of cellulose was performed using a titanate coupling agent that was incorporated in PLA by casting and melt extrusion [87]. The hydrophilicity of cellulose was reduced by surface modification, which made the cellulose compatible with the hydrophobic PLA matrix by reducing the water intake of the modified cellulose compared to unmodified cellulose. The modified cellulose increased the crystallinity of the composite with increased storage modulus, indicating better mechanical properties.

**Figure 4:**
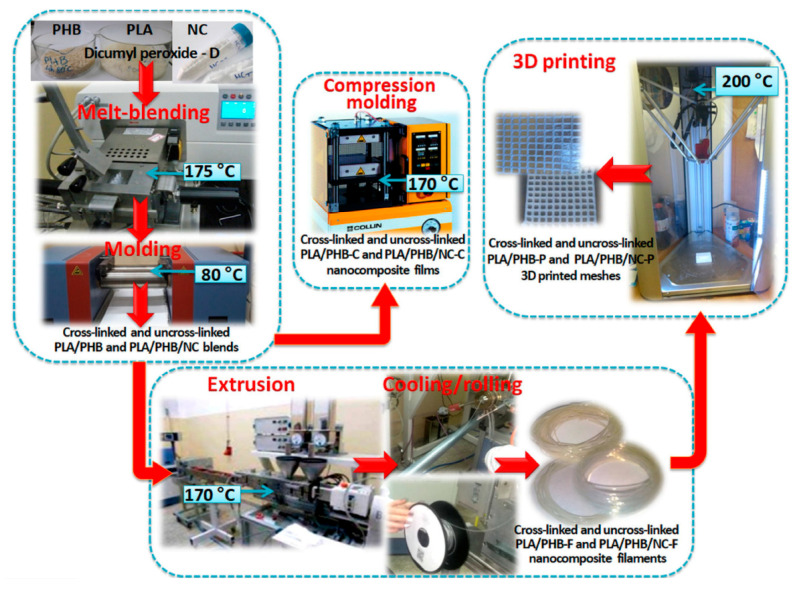
Schematic representation of PLA/PHB blends and nanocomposites preparation and processing of films, filaments, and 3D-printed meshes [90].

The other type of surface modifier that can be used is PEG that acts as a plasticizer to nanocellulose as it can promote uniform adhesion and dispersion of nanocellulose in the PLA matrix. Addition of nanocellulose was able to improve the composite thermal stability, whereas nanocellulose/PEG addition improved the crystallization rate, which is vital during printing for the buildability of printed products [88]. Furthermore, 3,6-dimethyl-1,4-dioxane-2,5-dione as L-lactide monomer was surface grafted on CNF also was able to improve the homogeneity in the PLA matrix, and it was found that the crystallinity of the extruded filaments increased about 100% [89]. On the other hand, PLA, poly(3-hydroxybutyrate), and CNC crosslinked with dicumyl peroxide was investigated for its properties through various fabrication techniques, as shown in Figure 4 [90]. While the dicumyl peroxide addition improved the homogeneity of CNC in the polymer matrix and enhanced the interfacial adhesion between the polymers. Incorporation of CNC was essential to maintain the thermal stability and delaying the degradation process during the extrusion, compression molding, and 3D printing, as low thermal stability was observed in the composite without CNC. The composites that were processed as filaments demonstrated the best thermal and mechanical properties compared to compression and 3D printing owing to better surface grafting of CNF and compatibility in the polymer matrix. 

Pickering emulsion approach is also a new method to incorporate TEMPO oxidized BC into PLA for a better dispersion of cellulose in polymer matrix [91]. Upon investigation of the crystallinity of the matrix subjected to 1.5% of TEMPO oxidized BC, the crystallinity increased by 2.16 times compared to PLA. The mechanical properties of the composite also increased tremendously due to the better dispersion of nanocellulose in the polymer matrix. This approach can also be used for other types of nanocellulose, particularly for 3D printing of biodegradable products.

Some types of cellulose do not require surface grafting; for example, hydroxypropyl methylcellulose (HPMC) derived from vegetable fibers can be incorporated readily in commercial polymer owing to the presence of the methyl group that can enhance the interfacial adhesion in the polymer matrix [92]. The mechanical strength of the 5% HPMC/PLA filament had tensile strength comparable to PLA with the contact angle enhanced up to 30 °C, and the improved thermal properties can pave the way for application of this composite for FDM printing in biomedical applications.

Biodegradable cellulose-based filaments without the presence of commercial polymers are essential for many applications, such as biomedical engineering. Cellulose-based filaments containing 50% CA and 50% cornstarch were extruded using a single-screw extruder, and the printing was carried out at the extrusion temperature between 230 and 240 °C with different flow rates of 80% and 90%. The sample heated at 230 °C with 90% material flow showed the best properties due to the lower voids present, better structural homogeneity, and good interlayer adhesion; in addition, higher printing temperature also led to poor adhesion and degradation of the sample. This method will be essential in the manufacturing of medical products for drug delivery and tissue engineering as it is highly biocompatible [93]. 

Furthermore, post-processing of the 3D printed cellulose composite part can be considered to match the industrial standard applications, as it can enhance the mechanical properties. The crystallinity of the printed part can be enhanced up to 200% through post processing if compared to neat polymer matrix, which will enhance the mechanical and thermal properties of the sample [89]. Another study on the 3D-printed product proved that the incorporation of grafted CNF with post-processing by annealing improved the static flexural modulus of the printed product by 151.94 times at 70% for 3% composite compared to the neat PLA without annealing [94]. Chemical post-processing can also be considered to reinforce the commercial polymer, as impregnation of CNC in methacrylate resin on an ABS printed sample was able to enhance the mechanical properties of the printed part [95]. Interfacial adhesion was improved upon impregnation, as indicated in the morphological analysis. The mechanical and thermal properties of the sample improved through the reinforcement of CNC and better dispersion demonstrated isotropic behavior. Table 3 reviews the typical reinforcement of cellulosic materials in commercial thermoplastic filaments for the FDM printing technique.

### 3.3. Vat Photopolymerization

Apart from FDM, VP has also been extensively improved through the development of liquid photopolymer resin due to the accuracy of the printed product, even though the method is comparatively longer than the extrusion technique. Research on the incorporation of fillers in resin is increasing in recent years, and one of the main approaches is cellulose-based fillers. The purpose of the incorporation is to improve the mechanical properties and thermal stability of the printed product. The compilation of the recent activity on the addition of cellulose-based polymer in photopolymeric resins is simplified in Table 4.

Cellulose that possesses hydrophilic properties can be homogenized effectively in the polymer matrix that is hydrophilic. For example, the addition of CNC in the photoinitiation of polyethylene diacrylate (PEGDA) improved the mechanical and thermal properties of the printed product [107]. Due to the hydrophilicity of CNC and PEGDA, the interlayer adhesion improved with the addition of 0.3% CNC, thus enhancing the tensile strength by 100% compared to the neat PEGDA.

Similar to FDM, surface grafting of cellulose has to be done to incorporate cellulose in hydrophobic polymer matrix. Incorporation of CNF that is surface grafted with reduced graphene oxide (rGO) and printed in SLA printer was able to enhance the tensile stress by 37% and nanoindentation hardness by 129% compared to neat polyurethane resin [17]. Morphological analysis showed the grafting of rGO in CNF nullified the hydrophilicity of CNF and improved the dispersion of CNF in the polyurethane matrix, whereas the incorporation of non-grafted CNF in polyurethane matrix displayed voids on the printed surface due to surface incompatibility of hydrophilic CNF and hydrophobic polyurethane. Lignin-coated CNC can also be considered as it was able to enhance the mechanical strength and thermal stability of photocurable methacrylate resin [108]. The addition of 0.1% lignin-coated CNC was sufficient enough to enhance the tensile strength and modulus with further increment of filler reduced the mechanical properties. A post-curing step of heating at 120 °C can be considered, as it further enhanced the mechanical properties due to the esterification of the resin matrix and lignin-coated CNC. 

**Figure 5:**
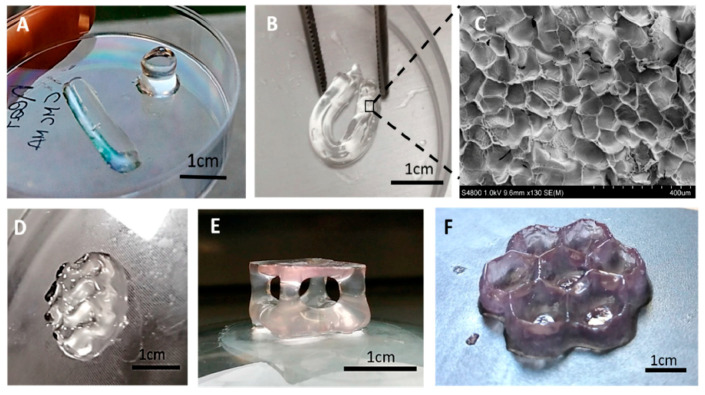
3D-printed M-CMC hydrogels: (**A**) Simple cylinders and parallelepipeds (solvent: water); (**B**) The hydrogel exhibited good flexibility and handleability; (**C**) SEM analysis performed on the freeze-dried hydrogel; and 3D objects printed from water (**D**) and culture medium solution (**E,F**) [109].

The recent development for cellulose based printing using VP technology is the development of methacrylated carboxymethyl cellulose bioink for biomedical application, as shown in Figure 5 [109]. CMC modified using methacrylate anhydride and added with photoinitiators to print complex geometries that possess high mechanical properties showed advancement in the development of VP inks, which are suitable for tissue engineering and, most importantly, biodegradable.

**Figure 6:**
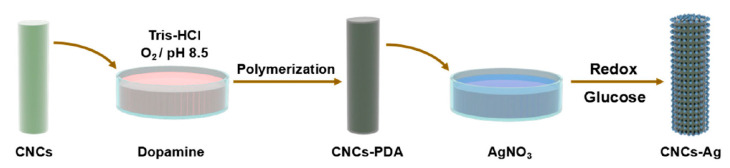
Synthetic route of nanocrystalline cellulose-silver (CNC-Ag) composite [110].

In addition, silver nanoparticles loaded with CNC were dispersed in polymethyl metharcylate to investigate the reinforcement in terms of mechanical and antimicrobial activities [110]. CNC grafted with dopamine and loaded in silver nanoparticles, as shown in Figure 6, improved the dispersion of silver nanoparticle–CNC composites in the polymer resin, as observed in its morphology. Incorporation of 0.1 wt.% of silver nanoparticle–CNC in the resin enhanced the mechanical strength of the composite by 12% as compared to neat resin. The antimicrobial characteristics of the composite was enhanced significantly by the incorporation of grafted CNC with silver nanoparticles compared to neat resin.

### 3.4. Liquid Deposition Modeling

As cellulose solution has the shear-thinning property, the solution can be readily used for ME printing technique, which is usually known as direct ink writing for liquid deposition modeling (LDM). The product printed with liquid cellulose should retain the shape after printing; thus, viscosity is essential, which is directly related to the concentration of cellulose and the shear rate applied. High mechanical strength of the printed part is vital to maintain the printed shape; therefore, increasing the concentration of cellulose can improve strength and reduce shrinkage, which consequently reduces the accuracy and smoothness of the printed part.

The design flow chart of any bioink is well-demonstrated in this study, where the prepared solution must initially satisfy the rheological behavior [114]. The concentration can be altered if the parameter does not satisfy the rheological requirement. The extrusion pressure and printing speed will be altered to obtain optimum printing line width. Cross-linking is studied to produce a solidified product and the agent can be varied to obtain a stable medium. Moreover, the sterility of the bioink is studied with cell proliferation to successfully produce bioink that is suitable for biomedical applications. The models can be altered to suit the application and desired results. 

Cellulose solution with smaller particles can be extruded through small-sized nozzles and maintained in the desired shape after printing. One of the earliest demonstrations of cellulose printing was the dissolution of cellulose in ionic solution, 1-ethyl-3-methylimidazolium acetate, and the results show that cellulose could be printed at low concentrations with a micro-sized dispensing nozzle passing through six layers [115]. The coagulation after printing obtained using a non-ionic solvent was able to regenerate the cellulose, thus acquiring a stable structure.

The other viable option for cellulose liquid printing is the dissolution of CA in acetone prior to printing and solidification of the printed part through evaporation [116]. The dissolution of the process enhanced the rheology of printing solution and the extrudability with the printing is accurately observed through the complex product of eyeglass frames and a rose. The hydroxyl bonds of cellulose can be restored with the removal of acetyl groups from the acetone. Surprisingly, these printed products had a tensile strength of 45 MPa, which is comparable to commercial polymers such as PLA and ABS, making it a favorable option for the commercialization of biodegradable material or cellulose-based printing.

Cellulose can also be printed readily without dissolution by the condition of optimum rheology and particle size to have smooth extrusion. CNC gels at different concentrations ranging from 11.8 to 30 wt. % in water were printed in an ordered structure with controllable porosity, printing resolution, and inner pore structure [117]. The shear-thinning behavior of nanocellulose allowed smooth extrusion, and high viscosity allowed a stable structure to be printed. The bowl structure printed using 20 wt. % CNC with different nozzle inner diameters showed different smoothness and shape deviations. The printing time required was compensated to achieve better print quality and smoothness as the difference was almost six times longer when the printing resolution was improved from 500 to 200 μm. The mechanical properties of the CNC aerogel were enhanced by the cross-linking with polyamide–epichlorohydrin, and an increment of Young’s modulus by 27% was observed. The potential of this printing method can be further studied as the method will be beneficial for tissue engineering applications with dual pore structures.

Other than CNC, CMC powder dissolution in water at the concentration of 45 wt. % and dispensed in a 30-μm nozzle successfully printed five square layers with stacking ability, and the total product height was 43 μm [118]. A low concentration of CMC printing resulted in ink smearing, and the line width printed was bigger than the nozzle, which affected the accuracy and smoothness of the printed products. The printing profile results in this study can be used for the fabrication of CMC products to be applied in tissue engineering. Further improvement of printing parameters needs to be conducted to enhance the resolution of the printed products. 

Furthermore, CNF suspension can also be printed at a lower concentration of 0.91 wt. % on a heated bed by controlling the extrusion pressure and printing speed [79]. CNF with the shear-thinning property extruded at the pressure of 172 kPa had an optimum flow rate of CNF printing. The variation of heated bed temperature also influenced the printed line width due to water evaporation, and a gel-like structure was formed. The hardness and tensile strength of the CNF printed product were 590 and 72.6 MPa, respectively, which can be adopted for biomedical engineering applications, as it managed to print CNF with mechanical properties comparable to CNF films in a much shorter time. 

**Figure 7:**
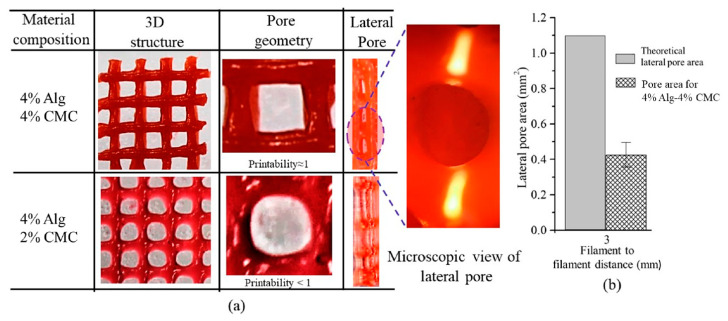
(**a**) Qualitative; and (**b**) quantitative tests for lateral pores [119].

Cellulose composited with different materials is also being studied intensively as cellulose provides the strength and shape fidelity while the fillers are used to have certain functionalities. Figure 7 shows that a study proved that 4% sodium alginate with 4% CMC has good extrudability and shape fidelity after printing as well as that a lower concentration of CMC did not have good printability. The printed sample was used in cell proliferation studies and determined to have comparable viable cells with alginate after 23 days, proving that CMC improves alginate printing without affecting alginate properties [119].

**Figure 8:**
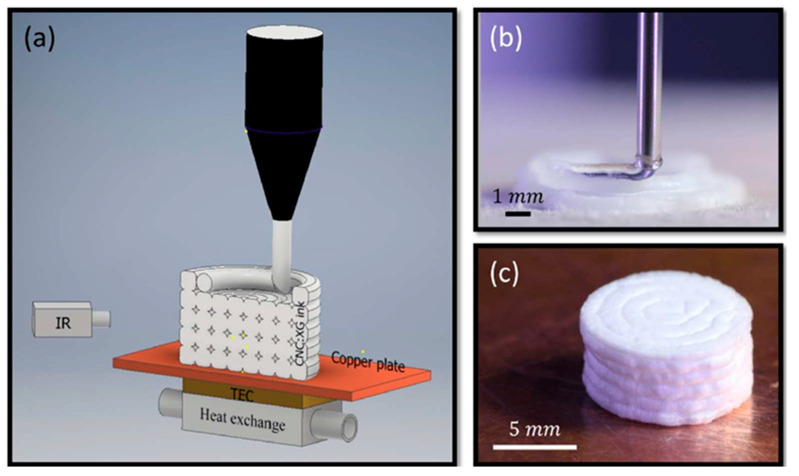
(**a**) Schematic illustration of the DCW setup; (**b**) photograph of the DCW printing process; and (**c**) photograph of the final aerogel [120].

Besides that, CNC with xyloglucan at a ratio of 100:1 (<4 wt%) can be printed using direct cryowriting (DCW) on a build plate that has freezing capability to build solid structures, as shown in Figure 8 [120]. Xyloglucan acts as a binder that enhances the extrudability of CNC, thus providing better shape fidelity of the printed structure. The impressive part of this formulation and cryoprinting method that can be adopted for cellulose-based printing is that a complex vase structure can be printed without support.

The LDM technique of commercial PLA with CA at different ratios was applied to determine the suitable viscosity for extrusion and the ability to maintain the shape fidelity after printing. Overall, 70% PLA and 30% CA were determined as the optimum combination, as the rheological behavior was found to have shear-thinning properties desirable for smooth extrusion. The increase of CA showed a detrimental effect on the extrusion, as the repulsive force present between CA and PLA reduced the storage and loss modulus. The optimum printing parameters were determined to be a print speed of 16 mm/s and extrusion pressure of 15 psi, which resulted in the printed line width of 250 μm [121]. 

BC with alginate printing has been done research immensely for applications such as fabrication of tissue scaffolds, antimicrobial composite with different additives such as copper and nanoclay [122,123]. It was found that 70 wt. % BC and 30% wt. % alginate were suitable for printing due to the shear-thinning property and ability to have a dimensionally accurate structure after cross-linking with calcium chloride [122]. The incorporation of BC helped to maintain the shape fidelity, as pure alginate printing shrank upon cross-linking. Table 5 reviews the studies done on liquid deposition of cellulose-based materials using various printing techniques and materials.

### 3.5. Applications

Instead of using synthetic polymers as materials for 3D printing, cellulose-based polymers are effective as the polymers are derived from natural resources, which can reduce the impact of fossil fuel depletion. Cellulose has several distinct properties, such as biocompatibility, biodegradability, surface grafting, and biodegradability, which are useful for many applications, such as medical, textile, tissue engineering, pharmaceutical, and electronic applications. The utilization of 3D-printed cellulose materials for various applications is reviewed in the following sections. 

#### 3.5.1. Biomedical Engineering

3D printing technology has been extensively used in medical applications, especially in drug delivery, tissue engineering, and wound healing. A recent study of 3D-printed nanocellulose double laminated with calcium carbonate was found to have controlled drug delivery of anticancer drug over the duration of 24 h, which is a good finding to reduce the side effect of drug burst release [138]. The drug release rate of the double laminated composite was up to 89.88% ± 0.37%, comparable to the drug release rate of porous calcium carbonate up to 91.37% ± 0.16%, thus achieving the objective of using nanocellulose as a drug carrier in a solution that has similar properties to the colon.

Carbamazepine drugs usually used to treat colonic seizure disorder were dispersed in ethyl cellulose and hydroxypropyl cellulose, and then printed as tablets to investigate the controlled drug delivery properties [139]. 3D-printed tablets showed a more controlled release of drugs compared to the 3D filaments containing carbamazepine, due to the high surface area exposure of the filaments, and the solution could penetrate the filaments deeper. Ethyl cellulose with a ratio of 2:1 to hydroxypropyl cellulose showed a zero-order controlled drug release profile for a period of 24 h; thus, this method can be studied for controlled drug release for some low dose drug treatments.

The optimization of the viscosity of polyurethane with hydrophilic properties was carried out for 3D printing by the addition of nanocellulose and triethylamine. The cell growth was better in the polyurethane/nanocellulose composite; thus, the condition is favorable for tissue engineering with the features of better mechanical properties and structural ability [140]. The viable cell rate in polyurethane/nanocellulose composite after a fortnight was 537%, while that of polyurethane/polyethylene oxide composite was about 300% owing to better cell attachment to the interior and surface of the fibers.

In a previous study, 95% PCL as a biodegradable polymer was added to 5% BC, and the mixture was printed to evaluate the extrudability and cell growth on the specimens [141]. After 72 h, the cell growth was observed and the viable cells were higher by 155% compared to the control, and the PCL-BC printed scaffold enhanced the cell proliferation through the 3D printing method. 3D printing is essential as the process can produce a proper scaffold and integrated structure that affects cell growth and alignment. The printing of CNF and alginate with the cross-linking of CaCl_2_ at different concentrations was studied to determine the best printing condition without shrinkage [142]. Cross-linking with a higher concentration of CaCl_2_ reduced the water absorption capacity of the sample, and also reduced the shrinkage of CNF/alginate composite. This study shows a positive finding that can be used in wound dressing application at the alginate concentration of 20 wt. % relative to CNF due to excellent water absorption and better structural ability.

The wound healing property of 3D-printed BC with chondroitin sulfate and hyaluronic acid was evaluated on a patient diagnosed with diabetic foot wound [143]. After applying the dressing on alternate days for one month of treatment, the study showed a positive result based on the reduction of the wound area and recovery at the surrounding area. Four months of treatment showed complete healing with minimal scar that can be concealed over time. This is a promising method for diabetic wound healing applications as BC is a promising material for cell growth. CA of 25 wt. % with a rheological modifier (i.e., glycerol) could be printed at a very high resolution of 99–608 μm with the optimization of extrusion pressure, printing speed, and extrusion nozzle diameter [144]. This approach can be used in the formulation of medical applications that require a high-resolution printed product. CA has good tissue engineering properties with high mechanical properties and the material is able to imitate the cell matrix for cell proliferation studies.

Cellulose-based 3D printing materials are emerging in biomedical applications, especially in controlled drug delivery, wound healing, and cell growth. In vivo studies are continuously conducted to overcome obstacles and further improve cellulose-based 3D printing materials. There is a small gap for this printing technique to be readily used for patients, but it will require approval for application on humans.

#### 3.5.2. Electronic Engineering Applications

Artificial intelligence robots are developing rapidly in recent years to ease human-to-machine interaction, hence driving the development of electronic sensors for the detection of signals from humans and processing the signals into the language that a machine can interpret. Cellulose-based 3D printing materials are important in the development of electrical engineering applications by the integration with certain metals to acquire stronger mechanical properties and obtain the desired functions. 3D printing will assist in the manufacturing of complex objects that are difficult to be printed through traditional manufacturing processes.

A humidity sensor was 3D printed with the composite of CA and aluminum to detect the humidity of sweat in a contactless manner [145]. The sensor was printed with a programmable response that could bend at 45 °C and would connect the circuit in the presence of a finger, which is the stimulus of humidity to trigger the alarm. This is a novel method that can be used in health monitoring and human–machine interaction applications.

The 3D printing of silver nanowire as a conductive material and CMC as a rheological modifier was performed to produce a lithium battery with an anode, a cathode, and a separator [133]. Increased conductivity was observed with the increasing concentration of silver nanowire, and the solid content of CMC/silver nanowire was fixed at 25 wt. % to obtain the desired viscosity for printing. The voltage of the cellulose-based printed battery was determined to be 1.8 V, which agrees with commercial batteries and proves that cellulose-based electronics can become the next generation material. The electrical conductivity of CNF composite with the addition of lithium produced using 3D printing technology was evaluated [146]. CNF is essential in maintaining the structure after printing due to its high mechanical properties, and the shear-thinning properties assist in the smooth extrusion of the battery anode and cathode. The 3D-printed lithium-based anode and cathode with CNF exhibited a high specific capacity with the advantage of various designs through 3D printing.

The study of 3D-printed CNF/alginate-based hydrogel cross-linked with calcium ions was conducted to evaluate the electrical conductivity for organic electronics [147]. CNF/alginate hydrogel functionalized with poly(3,4-ethylenedioxythiophene) displayed high electrical conductivity and could store electrical charge. As cellulose is able to alter its form with water presence due to its hygroscopic nature, the electrical conductivity changes with respect to humidity, thus making cellulose suitable to be used as a humidity sensor. 

CNF/silver nanowire composite demonstrated better electrical conductivity compared to CNC/silver nanowire in the production of a biocompatible sensor [148]. CNF/silver nanowire composite demonstrated better electrical conductivity compared to CNC/silver nanowire in the production of a biocompatible sensor. 3D-printed cellulose-based composites are being investigated thoroughly for the printing of electrically conductive materials due to cellulose properties that can maintain the shape fidelity and change the structure based on the surrounding conditions. 

#### 3.5.3. Other Applications

3D printing of food applications using cellulose-based materials has grown remarkably in recent years with the development of bioprinters with multi-nozzles. Research has been continuously done on the rheology of the material in order to obtain printable paste solutions because 3D printing can be used in food applications to produce food with the desired shape and amount. CNF-based food printing was done with the homogenization of milk powder and starch, as a small concentration of CNF helps to maintain the printed food structure [149]. Cellulose powder was bound with xanthan gum in a study where sample consisting of 10 layers were printed [150]. Optimization can be done on the viscosity and heating parameters to create more complex structures to be used in food-based products using BJ printing technique.

As biomaterials are increasingly used in textile and fashion industries to replace petroleum-based products, 3D printing of cellulose-based products can be studied to customize products based on the demand and to create stronger products. CA consisting of a linear structure and acetoxypropyl cellulose was printed on woven cotton-based fabrics to study the viability of cellulose-based materials for textile applications [151]. CA alignment with cellulose molecules on the substrate resulted in better adsorption and interfacial adhesion compared to acetoxypropyl cellulose but both cellulose based materials can be studied for development of all cellulose-based textile applications. CNF plasticized with glycerol was studied to be used as a coating on woven cotton fabrics and found that CNF coating reduces the amount of fabric pigment needed while maintaining the mechanical properties [152]. CNF coating on fabrics can be fabrication of electronic textiles for medical and other applications that do not require washing.

Neat cellulosic nanomaterials demonstrated excellent oxygen barrier properties compared to cellulose composite materials, which highlights the potential of airtight packaging for food and pharmaceutical applications [153]. The application of high-resolution CNC ink can be further studied for packaging and to satisfy customers’ needs on the desired packaging shape using LDM [117].

## 4. Cellulose-Based Polymers in 4D Printing Technology

### 4.1. Cellulose-Based Responsive Materials

Cellulose-based materials possess remarkable properties as discussed earlier and the addition of smart polymers allows for better applications in some industries. The response of these materials toward a stimulus is promising, coupled with sustainable and biodegradable nature, which can diversify the range of applications of the materials. New applications in biomedical engineering have led to increased attention on the extensive development of cellulose-based smart materials. In this section, different types of stimuli of recently developed cellulose-based smart materials are reviewed.

#### 4.1.1. Heat Responsive

CNC and polyurethane composite produced through direct melt mixing are thermally sensitive and exhibit shape-memory properties [154]. The addition of phosphorylated CNC improved stiffness and shape-memory properties compared to neat polyurethane. The flat sample was heated to 70 °C and the temporary shape was fixed as a spiral part by twisting around the metal rod. The flat shape could be recovered in 20 s and after placing the sample in an oil bath at 70 °C. The addition of CNC improved the shape fixity of the composite by bridging the crystalline domains present in the composites. Hence, CNC and polyurethane composite have a huge potential to be used in biomedical applications. 

Surface grafting CNC with lower critical solution temperature (LCST) polymer within polyvinyl acetate matrix produced good mechanical and shape-memory properties [155]. The stiffness of the composite could be reversed when immersed in hot water due to the collapse of the LCST polymer, and the properties could be reversed again by immersing in cold water. Hence, the CNC-polymer composite can be altered to be stiffer at body temperature, and this feature is suitable to be used as reinforcing implants in biomedical engineering applications.

#### 4.1.2. Moisture Responsive

As cellulose is hydrophilic in nature, many cellulose-based materials responsive to water have been developed and applied in various applications. Cellulose nanofibrils (CNF) were utilized to produce thin films with the potential to be used as biometric actuators. Water vapor can be used to control the actuation of CNF that reacts to the difference of humidity level [156].

The produced thin films can bend upon contact with humid layers, which is attributed to the increase in the volume of the hydrated layer. The uneven increase in the volume of the opposite side of the films leads to the bending motion, and similar findings have also been observed in other studies [157,158]. CNC-based films have also been used as humidity sensors with 99.8% colorimetric response value in the application of intelligent packaging for moisture-sensitive products [159]. CNC-based films have also been used as humidity sensors with 99.8% colorimetric response value in the application of intelligent packaging for moisture-sensitive products.

#### 4.1.3. Light Responsive

Photo-responsive cellulose-based materials exhibit changes in properties when exposed to light. A sun exposure sensor was developed by printing a mixture of titanium dioxide, polyvinyl propylene, and food dye on a paper. The titanium dioxide particles act as a photocatalyst. When exposed to the sun, the titanium dioxide particles degraded the food dye present in the paper, hence resulting in discoloration. Further calibration was conducted to match the UV exposure time for several different skin types [160]. 

Photochromic molecules were dissolved in ionic liquid and coated on cellulose fibers through hydrogen bonding, and the light-responsive properties were investigated [161]. The prepared paper demonstrated reversible and rapid photo-responsive color changing behavior when irradiated with UV and visible light. This allows potential applications in photowritable and photoerasable displays and UV sensors.

Photo-responsive cellulose nanoparticle coating was developed through a combination of donor–acceptor Stenhouse adducts and cellulose stearic acid ester. The developed composite could be switched reversibly between hydrophobic and hydrophilic properties through exposure to visible light. The coating can be applied onto papers and allow the production of paper-based fluid timers [162]. The incorporation of CNC and 2-bromoisobuuturyl bromide into 6-[4-(4-methoxyphenyl-azo) phenoxy] hexyl methacrylate produced a photo-responsive composite that changes in color through the irradiation to light with the wavelength of 350 nm [163].

#### 4.1.4. Magnetic Responsive

Magnetically-responsive cellulose-based materials can be produced by the deposition of magnetite nanoparticles on cellulose paper sheets [164]. The paper was subsequently coated with polydopamine film, which acts as a link and then used as an immunoassay platform for detection. The material can be applied in medical applications due to low cost and easy manufacturing. Terbium-doped germanium borosilicate glass as a magnetic source was loaded into cellulose, and the incorporation with graphene oxide managed to fabricate a magnetic cellulose paper [165]. Homogeneous stirring and electrostatic interaction will disperse the magnetic and graphene oxide particles, therefore improving the mechanical and magnetic properties of cellulose-based papers. 

#### 4.1.5. Electrical Responsive

CNF films exhibited piezoelectric properties that could withstand high electric fields despite its porous structure [166]. The piezoelectric properties showed a significant increase after the introduction of electric field due to the high crystalline region with a large piezoelectric coefficient. The produced films exhibited permittivity of 3.47 and 3.38 when tested with dielectric potentials of 1 kHz and 9.97 GHz, respectively; hence, the films are suitable in applications such as piezoelectric sensors, energy generators, and actuators. A CNF/polydimethylsiloxane composite used to fabricate piezoelectric nanogenerators exhibited high output piezoelectric signals with the voltage of 60.2 V, which were able to power 19 blue LEDs and a charge capacitor up to 3.7 V [167]. This composite can be used in electronics and self-powered systems due to its high piezoelectric properties. Lower piezoelectric properties were observed when cellulose microfiber was used instead of nanocellulose due to the high amorphous region in native cellulose [168].

#### 4.1.6. PH Responsive

Functionalized CNC and polyethylene glycol-poly(ε-caprolactone)-based polyurethane composite were used to fabricate a pH-responsive shape-memory polymer. The functionalization of CNC using pyridine and carboxyl groups induced the pH responsiveness of CNC through the hydrogen bond interaction at different pH. The fabricated pH-responsive polymer can be used as sensors, actuators, and biocompatible materials in biomedical engineering. Oxidized CNC can also be used in the fabrication of pH responsive sensors since the material shows reversible color changes at different pH values [169]. These characteristics assist in the development of bio-based sensors for chemical detection and intelligent packing materials.

### 4.2. 4D Printing of Cellulose-Based Responsive Materials

The merging of 3D printing technology and cellulose-based smart materials will be able to fabricate 4D-printed cellulose-based materials that can change shape over external stimuli. Many types of cellulose that are responsive to various stimuli reviewed in the previous section are suitable for the production of 4D cellulose materials. As cellulose possesses many useful properties such as biocompatibility, biodegradability, high mechanical properties, and thermal stability, the material will be a driving factor for the fabrication of cellulose-based 4D materials for applications in tissue engineering and medical applications.

CNF-based printing that mimicked the plant cell structure was embedded in the acrylamide matrix [170]. Clay was added to improve the rheological properties of the ink to be printable and to maintain the shape fidelity of the structure. The printed structure was cured under UV light for the cross-linking of acrylamide monomer. Glucose was incorporated to minimize oxygen interruption in the UV curing process. As a result, cellulose fibrils were well aligned, hence making the printed structure to have controlled swelling and exhibited anisotropy that induced reversible shape change with water stimulus. Through the variation of printing parameters, this hydrogel can be used to create structures that can change into the desired shapes after immersion in water.

Other than that, CMC, hydroxyethyl cellulose (HEC), and cellulose fiber composite were synthesized to study the characteristics and suitability of 4D printing of water stimulus cellulose-based structure [171]. Petal design was used to evaluate the shape-responsive properties to water stimulus. The hydration of the sample resulted in a flat surface and the dehydration resulted in a petal shape; furthermore, the configuration was cyclical as the cycle could be repeated more than five times, hence demonstrating good shape-memory behavior. A similar approach of HEC, microfibrillated cellulose (MFC), and lignin cross-linked with citric acid also demonstrated the shape-memory properties as the material became soft, expanded, and heavier after coming in contact with water stimulus [172]. Finite element method used in this study could be further explored for the fabrication of prototypes and desired structures for evaluating the shape-responsive properties. As this composite has higher mechanical properties and shape-responsive feature, the composite can be utilized in various biomedical applications.

Other than water stimulus, cellulose, and PVA composite cross-linked with glyoxal were 3D printed and it was found that the printed structure had shape-memory properties responsive to heat and moisture [173]. The T_g_ of the composite was 70 °C and its state could be changed by heating above the temperature. The shape memory of the printed tensile bars was tested by heating above T_g_ for temporary shape programming and cooling below T_g_ with the stress maintained. The desired printed shape was obtained after heating above T_g_ or immersing in water at 37 °C within 90 min. The developed method has potential applications in the biomedical industry because the method can be moisture- and heat-triggered to have shape-memory properties, as well as to improve biodegradability as cellulose-based waste materials can also be incorporated.

**Figure 9:**
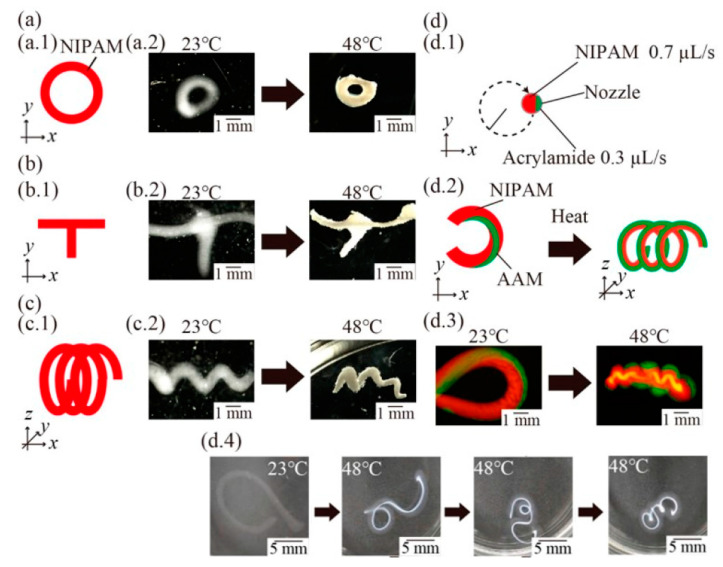
A demonstration of 4D printing: (**a**) schematic illustration and images of printed structures and heated structures with a rounded pattern; (**b**) schematic illustration and images of a fabricated T-shaped structure and a heated T-shaped structure with a cross point; (**c**) schematic illustration and images of a fabricated spring structure and heated spring structure with an internal gap; and (**d**) schematic illustrations of printing a C-shaped structure with multi-hydrogels (**d.1**) and its 3D deformation from the C-shaped structure to the spring-shaped structure. (**d.2**) Fluorescence images of the fabricated C-shaped structure (**d.3**) and the transformed spring structure obtained by heating. (**d.4**) Time-lapse images of the C-shaped structure [174].

Furthermore, stimulus responsive properties of N-isopropylarcylamide, CMC, arcylamide, and sodium alginate composite were studied using heat stimulus [174]. Various structures printed from the composite solution such as angulated and rounded patterns, T-shaped, and spring-shaped structures. The structures could undergo a transformation in different ways such as spring structure could be formed from C-shaped structure, as shown in Figure 9. 4D printing of cellulose-based materials will be essential in biomedical engineering applications, especially for tissue-based applications and soft robotic applications. Table 6 reviews the studies done on 4D-printed cellulose-based materials using LDM technique.

## 5. Summary, Conclusions, and Future Trends

AM is a growing industry, and continuous research is being done to improve the technology and materials available. The development of materials with composites enables the fabrication of products with better mechanical properties, which can be fine-tuned according to the demand. AM enables the fabrication of customized products according to customer’s design requirement and offers design flexibilities. AM plays a vital role in reducing the burden of traditional manufacturing processes in the fabrication of prototypes and testing the properties of the printed products.

Currently, more sustainable materials are preferred; as most of the AM materials are made from non-renewable resources, naturally-derived materials are adapted, and new studies are being done to improve interfacial adhesion and voidance for the application in various fields. Some applications such as wound healing or drug delivery cannot adapt AM until natural polymers are adopted as they are biocompatible. The proof of concept and in vivo studies are being continuously done in order to use natural-based materials for AM so that the applications can be broadened, where the materials will be the future trend of material development.

Cellulose, which is mainly derived from lignocellulosic biomass, possesses various useful properties; hence, more studies should be done to incorporate cellulose in AM. The potential of cellulose materials in AM is yet to be fully tapped, even though major developments can be seen in recent years with studies being conducted for various applications. Surface grafting of cellulose can nullify the hydrophilicity of cellulose, and hence voidance and interfacial adhesion can be improved. Further research should be carried out continuously to improve the mechanical properties so that AM-fabricated products will have better mechanical properties than traditionally-manufactured products. The increment of cellulose percentage in 3D printing filaments is also essential to improve the biodegradability of filaments and reduce the burden on petroleum resources, as well as to protect the environment as petroleum-based materials emit unpleasant odors.

Liquid deposition modeling of cellulose materials should also be further studied as the rheological properties of cellulose and cellulose derivatives favor the extrusion process. A high concentration of cellulose should be incorporated due to its high mechanical properties, and the structure of the printed part can be maintained after printing. Studies have shown that in vivo 3D-printed BC was able to cure diabetic wounds within four months, and many more studies should be done to present further proof. Cellulose materials can be synthesized with the desired properties according to the needs of applications and incorporated in AM for various types of applications with the developing technology.

4D printing of bio-based materials is a newer technology compared to other AM-based technologies. Cellulose-based 4D printing materials are majorly developed based on water and heat stimuli. Further studies should be done on other stimulus-responsive cellulose-based materials for 4D printing, as vast cellulose smart materials are available and developed continuously. The development of cellulose-based 4D printing will contribute majorly in tissue engineering and drug delivery application, which can also change the prospect of the healthcare industry in the treatment of patients by using AM technologies.

## Figures and Tables

**Table 1:** The advantages AM technologies of polymer-based materials and its potential applications.

Technology	Material	Printing Resolution (mm)	Advantages	Applications	Ref.
Material Extrusion (ME)	Thermoplastic polymers	0.05–0.6	Ease of use, low cost, wide availability of printers	Rapid prototyping, soft robotics, tissue engineering	[35]
Vat Polymerization (VP)	Photocurable resins	0.025–0.1	Low cost, high resolution, smooth printing surface	Tissue engineering, dental products, electronic insulators	[36]
Binder Jetting (BJ)	Ceramics, metals, Biomaterials	0.3–0.5	Fast printing, bigger build volume	Prototypes, casting patterns	[37]
Powder bed fusion (PBF)	Metal, polymers	0.08–0.25	High resolution, good quality	Electronics, aerospace, biomedical engineering	[38]
Material Jetting (MJ)	Photocurable polymers	0.016–0.060	Very high resolution, multicolor printing	Rapid prototyping, casting patterns	[39]
Directed Energy Deposition (DED)	Metal powder, polymer composite	0.25–0.3	Low manufacturing cost, high strength product	Aerospace, manufacturing	[40]
Sheet Lamination (SL)	Polymer, metal, ceramics	0.1	Low cost, wide available materials	Electronics, paper production	[41]

**Table 2:** The modification approaches on the biodegradable of PLA.

Filler	Filler Fraction (%)	Composite Tensile Strength (MPa)	Difference (%)	Ref
Modified carbon fiber	34	91.0	+225.0	[52]
Carbon fiber	28	61.4	+36.8	[53]
Graphene nanoplatelets	10	40.2	+27.2	[54]
Rice husk	20	53.0	+18.3	[55]
Ceramics	40	43.2	+1.9	[56]
Poplar fibers	20	54.0	0.0	[57]
Copper	40	40.3	−4.9	[56]
Aluminum	40	40.2	−5.1	[56]
Calcium carbonate	20	37.0	−17.8	[58]
Lignin	40	45.65	−21.9	[59]
Wood fiber	60	13.49	−63.9	[60]
Cork granules	50	10.4	−82.6	[61]

**Table 3:** Recent findings on the cellulose composited in thermoplastic filament for FDM extrusion.

Polymer/Cellulose Composition (wt%)	Extrusion Technique	FDM Printer	Nozzle Temp. (°C)	Nozzle Diameter (mm)	Improvements.	Ref.
73.5% PLA, 24.5% PHB, 1% CNC, 1% dicumyl peroxide	Twin-screw	WASP Delta 2040 Turbo 2	200	0.4	Mechanical properties and thermal stability	[90]
93% PLA, 7% Hydroxypropyl methylcellulose	Single-screw	Ender-3S	200	0.4	Thermal properties and contact angle	[92]
70% PLA, 25% recycled PLA, 5% MCC, 0.5 phr epoxy-based chain extender	Twin-screw	LulzBot TAZ 6	200	0.5	Tensile strength, modulus and Izod impact strength	[96]
95% PLA, 5% Hemp powder	Single-screw	Ultimaker 3	180	0.4	Elastic modulus, tensile strength	[97]
95% ABS, 5% CNC/Silica nanohybdrids	Twin-screw	S1 Architect 3D	235	0.3	Reduced warping, tensile strength, and layer adhesion	[98]
90% Polycaprolactone, (PCL), 10% MCC	Single-screw	Prusa i3	210	0.4	Mechanical strength and cell proliferation	[99]
80% Polypropylene (PP), 20% cellulose waste materials	Twin-screw	Lulzot Taz 6 MEAM	220	0.8	Storage and elastic modulus	[100]
95% ABS, 5% Oil palm fibre	Single-screw	UP Plus 2	N/A	0.4	Tensile strength and elastic modulus	[101]
80% PVA, 20% CNC	Single-screw	Sharebot Next Generation	230	0.35	Tensile strength and thermal properties	[102]
90% ABS, 10% Lignin coated CNC	Single-screw	Solidoodle 3	210	0.35	Mechanical and thermal properties	[103]
70% PLA, 30% CNF	Single-screw	Solidoodle 3	180	0.35	Tensile strength and elastic modulus	[104]
87% PLA, 13% polybutylene adipate terephthalate (PBAT), 40 phr Hemp hurd	Twin-screw	3D da Vinci 1.0	200	0.4	Flexural modulus and dimensional accuracy	[105]
50% Poly (ε-caprolactone), 50% cocoa shell waste	Single-screw	Prusa i3 Hephestos	120	0.3	Mechanical and thermal properties	[106]

**Table 4:** The compatibility of cellulose-based biopolymer as a filler in photopolymeric resins.

Cellulose Composition	3D Printer	Printing Parameter	Solidification Method	Potential Application	Ref.
Polyurethane aryclate, CNF-rGO, CNF-PEG	Wanhao Duplicator D7 Plus	UV light of wavelength 405 nm	UV curing	Bio based resin	[17]
Polymethyl methacrylate (PMMA), CNC-Silver Nanoparticles (CNC-AgNPs)	Envision TEC	Layer thickness 100 μm, 4.4 s exposure time, UV intensity 2500 μm/cm^2^	UV curing	Dental restoration material	[110]
CNC, methacrylate resin	Form 1+	N/A	Photocuring and heating	Electronic, engineering and tissue engineering	[111]
Ethyl cellulose macromonomerm resin-based monomer	Creality, LD 001,	N/A	Photocuring	Flexible electronic materials	[112]
CNC, PEGDA, 1,3-diglycerolate diacrylate (DiGlyDA)	DLP 3D printer	Layer thickness 100 μm, 4.0 s exposure time, UV intensity 18 mW/cm^2^	UV curing	Biomedical application	[113]

**Table 5:** Review of cellulose matrix as 3D printing hydrogel using LDM technique.

Cellulose Composition	3D Printer	Printing Parameter	Solidification Method	Potential Application	Ref.
Dialdehyde CNC, gelatin	Bio-Architect	Nozzle 0.21 mm, Extrusion pressure 100–250 kPa, Print speed 10–40 mm/s	Crosslinking with Ca^2+^	Tissue engineering	[124]
CNF, Alginate	Regemat3D Designer	Nozzle 0.58 mm, Flow speed 3.0 mm/s	Crosslinking with CaCl_2_	Tissue engineering	[125]
Bacterial CNF, silk fibroin (SF)/gelatin composite	3D Bioplotter	Nozzle 0.41 mm, Extrusion pressure 1–2 bar, Print speed 3.0	Crosslinking with genipin	Biomedical applications	[126]
CNF, xylan-tyramine	3D bioprinter, RegenHU, Switzerland	Nozzle 0.42 mm, print speed 40 mm/s, layer height 0.4 mm	Crosslinking with H_2_O_2_	Clothes, packaging, health care products, furniture	[127]
CNF, CMC	Bioscaffolder 3.1	Nozzle 0.25 mm, Extrusion pressure 260 kPa, print speed 15 mm/s	Crosslinking with dehyrothermal treatment (DHT)	Bone tissue engineering	[128]
CNF, carbon nanotubes,	Fisnar F4200n,	Nozzle pressure 345 kPa, Print speed 10 mm/s	Solvent exchange with ethanol	Biomedical application	[129]
CNF, 2,2,6,6-Tetramethyl-1-piperidinyloxy	Custom built LDM printer	N/A	Oven treatment	Oil-in-water separation, electronic related application	[130]
CNC (20 wt%)	Custom built multi-material-multi-method 3D printer	Nozzle 0.4 mm Extrusion pressure kPa, Print speed 40 mm/s	None	Support during 3D printing	[131]
CNC, sodium alginate, gelatin	Ultimaker with Discov3ry Complete	Print speed 30 mm/s, printing temperature 26 °C	Crosslinking with CaCl_2_	Cartilage regeneration application	[132]
CMC, silver nanowire, lithium iron phosphate/lithium titanate	Delta FDM 3D printer integrated with Discov3ry Extruder	Nozzle 0.84–1.54 mm, print speed 0.7–1.2 mm/s, Extrusion flow rate 0.0002–0.00083 mL/s	Drying	Conductive nanocomposites	[133]
CNC	ABL 900010,	Nozzle 0.41 mm, extrusion pressure 200–400 kPa, print speed 10–20 mm/s	Drying	Cellular architectures	[134]
CNC, 2-hydroxyethyl methacrylate monomer, polyether urethane acrylate oligomer, photoinitiator	3D Discovery, RegenHU Ltd., Switzerland	Nozzle 0.41 mm, extrusion pressure 300–400 kPa, print speed 10 mm/s	Photocuring	Cellular architectures	[134]
Cellulose, N-methylmorpholine-N-oxide (NMMO)	3D Bioplotter	Printing temperature 70 °C	Coagulation in water (NMMO removal), freeze drying	Tissue engineering	[135]
Cellulose fibers, CMC	Prusa i3 with WASP extruder	Nozzle 0.7 mm, print speed 10–20 mm/s	Solvent exchange, Drying	Lightweight 3D printed material	[136]
CNF, carbon nanotubes, NaOH	RegenHU 3D Discov3ry	Nozzle 0.3 mm, Extrusion pressure 65 kPA, Print speed 10 mm/s	Drying (ambient condition)	Neural tissue engineering	[137]

**Table 6:** Review of 4D printed cellulose based materials using LDM technique.

Cellulose Composition	3D Printer	Printing Parameter	Solidification Method	Stimulus	Potential Application	Ref.
CNF, clay, N-isopropylacrylamide	ABG 10000, Aerotech	Nozzle 0.15–1.5 mm	UV Curing	Water	Tissue engineering and soft robotics applications	[170]
CMC, cellulose fibers, HEC, clay	Prusa MK2	Nozzle 0.8 mm, layer height 0.6 mm	Crosslinked with citric acid	Water	Tissue engineering applications	[171]
HEC, MFC, citric acid/hydrochloric acid, lignin	Modified TEVO Tarantula i3	Nozzle 0.55–4 mm	Crosslinking using citric acid/hydrochloric acid	Water	Biomedical application	[172]
MFC, PVA	3D Bioplotter, EnvisionTEC	Extrusion pressure 5.0 bar, print speed xy 400 mm/min, print speed Z 350 mm/min, layer thickness 0.67 mm	Crosslink using glyoxal solution	Heat and Water	Tissue engineering applications	[173]
N-isopropylacrylamide, CMC, sodium alginate, acrylamide	Custom built printer	Extrusion flow rate 1.0 μL/s, print speed 1.0 mm/s	Irradiation with UV light, then soaked in water	Heat	Environmental monitoring and medical applications	[174]
CMC-Na, clay, HEC	N/A	N/A	Crosslinking with citric acid	Water	Tissue engineering applications	[175]
CMC-Na, montmorillonite clay	N/A	N/A	Crosslinking with citric acid	Water	Tissue engineering applications	[176]
